# Rapid Diagnosis of Childhood Pulmonary Tuberculosis by Xpert MTB/RIF Assay Using Bronchoalveolar Lavage Fluid

**DOI:** 10.1155/2014/310194

**Published:** 2014-08-06

**Authors:** Qing-Qin Yin, Wei-Wei Jiao, Rui Han, An-Xia Jiao, Lin Sun, Jian-Ling Tian, Yu-Yan Ma, Xiao-Chun Rao, Chen Shen, Qin-Jing Li, A-Dong Shen

**Affiliations:** Key Laboratory of Major Diseases in Children and National Key Discipline of Pediatrics (Capital Medical University), Ministry of Education, Beijing Pediatric Research Institute, Beijing Children's Hospital, Capital Medical University, 56 Nan Li Shi Road, Xicheng District, Beijing 100045, China

## Abstract

In order to evaluate the diagnostic accuracy of the Xpert MTB/RIF assay on childhood pulmonary tuberculosis (PTB) using bronchoalveolar lavage fluid (BALF), we evaluated the sensitivity, specificity, positive predictive value, and negative predictive value of Xpert MTB/RIF assay using BALF in comparison with acid-fast bacilli (AFB) microscopy and *Mycobacterium tuberculosis* (MTB) culture for diagnosing childhood PTB using Chinese “composite clinical reference standard” (CCRS) as reference standard. Two hundred fifty-five children with suspected PTB were enrolled at Beijing Children's Hospital from September 2010 to July 2013. Compared with Chinese CCRS, the sensitivity of AFB microscopy, MTB culture, and Xpert MTB/RIF assay was 8.4%, 28.9%, and 53.0%, respectively. The specificity of three assays was all 100%. Xpert MTB/RIF assay could detect 33.9% of cases with negative MTB culture, and 48.7% of cases with negative AFB microscopy. Younger age (<3 years), absence of BCG scar, and contact with TB patient were found significantly associated with a positive result of Xpert MTB/RIF assay. In conclusion, Xpert MTB/RIF assay using BALF can assist in diagnosing childhood PTB much faster when fiberoptic bronchoscopy is necessary according to the chest radiograph.

## 1. Introduction

Tuberculosis (TB) caused by* Mycobacterium tuberculosis* (MTB) is one of worldwide infectious diseases. According to the estimation of the World Health Organization (WHO), there were an estimated 8.6 million new TB patients globally in 2012, including about 530,000 (6.2%) children less than 15 years old [1]. In TB high burden settings, childhood TB accounts for 15%–20% of total TB incidence [2]. In China, the 4th national TB epidemic survey showed that the rate of MTB infection in children between 0 and 14 years old was 9.0% [3] and the prevalence of active TB was 91.8 per 100,000 [4]. The gold standard of adult TB diagnosis is acid-fast bacilli (AFB) microscopy and MTB culture [5]. Although AFB microscopy is very fast and easy, less than 15% of children cases are sputum AFB microscopy positive [6]. In its turn, MTB culture needs 3-4 weeks, and it requires viable bacterium. Long time to culture MTB would delay the diagnosis and treatment of TB, and the specimens containing viable bacterium may be difficult to obtain, especially in patients having received anti-TB treatment. Due to the paucibacillary nature of childhood TB, its clinical diagnosis greatly relies on indirect symptomatic evidence. In China it is defined as “composite clinical reference standard” (CCRS) and involves the following 8 items [7]: (I) clinical manifestations including fever and cough for more than 2 weeks or gasping; (II) chest X-ray consistent with pulmonary TB; (III) contact with active TB patient; (IV) positive tuberculin skin test; (V) positive AFB microscopy or MTB culture using sputum, gastric lavage, or bronchoalveolar lavage fluid (BALF); (VI) response to anti-TB treatment; (VII) other excluded pulmonary diseases like pneumonia, lung tumor, lung cyst, and interstitial lung diseases; (VIII) pathology of lung tissue consistent with pulmonary TB. Besides meeting criteria I and II, patients meeting any 2 criteria of the items III, IV, VI, and VII are defined as clinical diagnosed.

Xpert MTB/RIF assay is an automatic molecular test based on seminested real-time PCR and molecular beacon technology targeting the* rpoB* gene. It can detect MTB and RIF resistance within 2 hours, which means the turnaround time is much shorter than MTB culture [8]. In December 2010, Xpert MTB/RIF assay was endorsed by WHO and then recommended that it needs to be evaluated in pediatric TB [9]. So far, only several studies about Xpert MTB/RIF assay focused on the application of Xpert MTB/RIF in children. The tested specimens included gastric lavage aspirates [10], sputum [11, 12], and stool specimens [13, 14]. Recently fiberoptic bronchoscopy is widely used to help in diagnosing and treating pulmonary TB, especially in those smear-negative cases and in cases who are difficult to cough up sputum, like children [15]. This study evaluated the diagnostic value of the Xpert MTB/RIF assay on childhood pulmonary TB (PTB) using BALF.

## 2. Methods

### 2.1. Ethics Statement

This study was approved by the Ethics Committee of Beijing Children's Hospital affiliated with Capital Medical University. The patients and parents/guardians on behalf of the children and adolescents included in this study were given information consent form and they all signed to participate in the study.

### 2.2. Study Population and Samples

**Figure 1:**
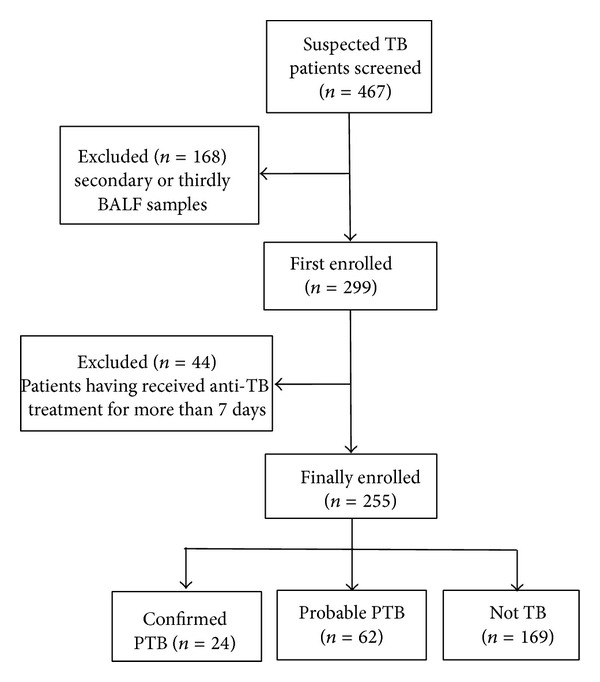
Study profile.

This study was conducted in Beijing Children's Hospital (Beijing, China) from September 2010 to July 2013. Children aged 18 years or younger were eligible for enrollment if they had been admitted to hospital, with suspected PTB having a cough for more than 2 weeks; a chest radiograph suggested to do routine fiberoptic bronchoscopy and with informed consents to do fiberoptic bronchoscopy. Children were excluded if they have received more than 7 days of anti-TB treatment and if informed consent was not obtainable. A total of 467 BALF specimens were collected from 299 enrolled suspected PTB patients and were stored at −20°C. Considering clinical information of all patients, 63 patients had more than one specimen each person; one sample was kept for every patient, excluding other 168 secondary or thirdly specimens from these 68 patients. And then 44 patients having received more than 7 days of anti-TB treatment were also excluded. Finally, 255 specimens from 255 patients were included in this study (Figure 1).

Classification of TB diagnosis was based on the CCRS criteria detailed above; combined with the classification of intrathoracic TB in children by Consensus from an Expert Panel [16], the suspected PTB patients were categorized into 3 groups: confirmed PTB patients; probable PTB patients; and not-TB patients (i.e., patients finally with established alternative diagnosis). Thus PTB patients included confirmed PTB patients and probable PTB patients.

### 2.3. Diagnostic Methods

The volume of BALF specimens varied from 0.8 to 4.0 mL. Every specimen was equally divided into two parts. One part was used for smear microscopy, MTB culture, and drug susceptibility testing (DST) of RIF, isoniazid (INH), ethambutol (EMB), and streptomycin (STR). The other part was used for Xpert MTB/RIF assay. Two different technicians performed these two parts in parallel and were blinded with regard to the results of other tests.

One part of specimen was send to Chinese Center of Disease Control and Prevention (Chinese CDC) to be tested directly and AFB microscopy (Ziehl-Neelsen staining) followed by processing with N-acetyl L-cysteine and sodium hydroxide (NALC-NaOH) [17]. Then the resuspended pellet was subjected to cultivation on both solid medium (Löwenstein-Jensen (LJ)) and liquid medium (BACTEC MGIT 960 culture). Either solid-culture-positive or liquid-culture-positive sample was considered as culture-positive. Culture-positive samples were confirmed to belong to MTB species by p-nitro benzoic acid assay [18] and were further subjected to indirect drug-susceptibility testing with MGIT SIRE.

The other part of the BALF specimen was used to perform Xpert MTB/RIF assay, which is a heminested real-time PCR method that targets the 81 bp region of the RIF-resistance determining region (RRDR) of the* rpoB* gene [8]. According to the manufacturer's suggestion, a 2 : 1 volume of sample reagent buffer (SR) was added to BALF, except for some samples (<1 mL) which were processed directly by adding SR up to 2 mL. The mix was vortexed for 20 seconds and left for 15 min. After that, 2 mL of the mix was added to the cartridge containing washing buffer, reagents for lyophilized DNA extraction and PCR amplification, and fluorescent detection probes (five overlapping the* rpoB* RRDR, one for sample processing control, as this assay contains lyophilized* Bacillus globigii* spores) [8]. The results of Xpert MTB/RIF assay were automatically generated within 2 hours and reported as MTB-negative or MTB-positive (with semiquantification) and RIF-sensitive or resistant. In case of reporting as “Invalid,” “No Result,” or “Error,” the specimen was considered as indeterminate.

### 2.4. Statistical Analysis

The sensitivity, specificity, positive predictive value (PPV), and negative predictive value (NPV) of AFB microscopy, MTB culture, and Xpert MTB/RIF assay were calculated against CCRS based on the single direct test run. The chi-square test was used in comparison of the sensitivity of AFB microscopy, culture, and Xpert MTB/RIF assay. The factors associated with positive rate of Xpert MTB/RIF assay in PTB patients were analyzed by two binary logistic regression analyses. All confidence intervals were two sided; 95% confidence intervals and *P* value < 0.05 were considered as statistically significant. IBM SPSS 17.0 software was used for statistical analysis.

## 3. Results

### 3.1. Descriptive Statistics

Of all 255 enrolled patients, 24 (9.4%) were confirmed to be PTB patients (7 smear-positive and 17 smear-negative), including 7 patients with positive liquid culture and negative L-J culture and 17 patients with positive L-J culture and positive liquid culture; 62 (24.3%) were probable PTB patients; 169 (66.3%) were not-TB patients, who finally were diagnosed as mycoplasma pneumonia. In all PTB patients, confirmed PTB patients accounted for 27.9% (24/86) and probable PTB patients accounted for 72.1% (62/86) (Figure 1). The mean age of the patients was 6.1 ± 3.68 years old with an age range of 0.3–15.3 years old. Two hundred and twelve (83.1%) patients had BCG scar; 27 (10.6%) patients had a reported history of TB contact. The characteristics of enrolled patients are shown in Table 1.

### 3.2. Diagnostic Test Results

In all results of 255 patients, the Xpert MTB/RIF assay results of 4 patients (3 probable PTB and 1 not-TB patients) were indeterminate, including 2 “Error” and 2 “Invalid” results. Thus the indeterminate rate of Xpert MTB/RIF assay was 1.6% (4/255). For the analysis of diagnostic accuracy, the data from these 4 patients were excluded.

Compared with CCRS, in all TB patients (confirmed and probable), the sensitivity of smear microscopy was found to be 8.4% (7/83) and that of MTB culture was found to be 28.9% (24/83), while the sensitivity of Xpert MTB/RIF assay against CCRS was found to be 53.0% (44/83), and the specificity was 100% (Table 2). The sensitivity of Xpert MTB/RIF assay was significantly higher than that of MTB culture (*P* < 0.0001). The positive and negative predictive values of Xpert were 100% and 81.2%, respectively, while those of MTB culture were 100% and 74.0% (Table 2).

In 83 CCRS positive patients including 24 confirmed PTB patients and 59 probable PTB patients, the sensitivity of Xpert MTB/RIF assay was 100% (24/24) for confirmed PTB patients, being 33.9% (20/59) for probable PTB patients. This means that Xpert MTB/RIF assay detected extra 33.9% PTB patients more than MTB culture. In 76 smear-negative PTB patients, the sensitivity of Xpert MTB/RIF assay was 48.7% (37/76); that is, Xpert MTB/RIF assay detected extra 48.7% PTB patients more than AFB microscopy (Table 3).

Regarding drug susceptibility testing (DST), of all 24 positive MTB culture samples, three samples did not have DST results because the strain failed to grow. Xpert MTB/RIF assay detected one case resistant to RIF correctly. This case was confirmed as MDR-TB by DST which also showed that this sample was resistant to INH, RIF, EMB, and STR. Other 20 samples were found to be susceptible to all tested drugs by DST and were found to be RIF-susceptible by Xpert MTB-RIF assay.

### 3.3. Factors Associated with the Positive Rate of Xpert MTB/RIF Assay

Further, the factors associated with the positive rate of Xpert MTB/RIF assay in all suspected TB patients were analyzed in 248 patients (Table 4). The factors included gender, age, BCG vaccination, and reported history of TB contact. The results showed that age was significantly associated with Xpert MTB/RIF assay; that is, the positive rate of detecting PTB patients younger than 3 years old was significantly higher than that of PTB cases older than 3 years old (*P* < 0.0001). The positive rate of Xpert MTB/RIF assay in patients with no BCG scar was significantly higher than that with BCG scar (*P* = 0.001), and the positive rate of Xpert MTB/RIF assay in patients having reported history of TB contact was significantly higher than that without reported history of TB contact (*P* = 0.010).

## 4. Discussion

For children with complicated pulmonary diseases, fiberoptic bronchoscopy can assist in diagnosing clinically; also Goussard et al. reported that the BALF culture yield was high in children with severe airway obstruction [19], although MTB culture from BALF has limitations to diagnose in children with uncomplicated pulmonary TB because taking BALF is much more invasive [20]. In our setting, all children need to do routine fiberoptic bronchoscopy according to their chest radiograph, and 28.9% of children who suffered from PTB had positive MTB culture, while 8.4% had positive AFB microscopy. Generally in developing countries, the low 10%–15% positive rate of the AFB microscopy in pediatric TB cases is accompanied by very low sensitivity of the MTB culture, since 20% to 80% of children who suffered from TB do not have positive MTB culture results [21]. And a retrospective study of pediatric TB cases in hospital also found the positive rate of AFB microscopy or MTB culture (either one was positive) was only 9.7% [22]. That suggests just using AFB microscopy and MTB culture to confirm childhood PTB is not nearly enough.

Because of low sensitivity of MTB culture in childhood PTB, CCRS was taken as the “gold standard,” and then the sensitivity of Xpert MTB/RIF assay can reach 53.0%, similar to that reported in the study of Nhu et al. (50%), which also took clinical diagnosis as “gold standard” [23]. Compared with CCRS, the sensitivity of Xpert MTB/RIF assay was significantly higher than that of culture and AFB microscopy. Xpert MTB/RIF assay can detect 33.9% more PTB patients than MTB culture and 48.7% more PTB patients than AFB microscopy, which is consistent with other studies conducted on children. Walters et al. found that Xpert MTB/RIF assay had 14% additional diagnostic yield because Xpert MTB/RIF assay had confirmed 2 cases with negative culture [24]. Chisti et al. reported the diagnostic yield of Xpert MTB/RIF assay was higher than culture in both induced sputum (7.6% versus 2.5%) and gastric lavage (5.1% versus 1.5%) in TB children with severe malnutrition and pneumonia [25]. Thus the above-mentioned studies have showed that Xpert MTB/RIF assay has a good application future in childhood TB.

In this pediatric TB study, all confirmed PTB patients were correctly detected by the Xpert MTB/RIF assay. With regard to the remaining probable PTB patients, the sensitivity of Xpert MTB/RIF assay was 33.9%. Because the probable PTB patients are all diagnosed clinically, it is urgent to find one method to diagnose such patients, and the Xpert MTB/RIF assay offers a way for their early and rapid diagnosis. Finally, the sensitivity of Xpert MTB/RIF assay in smear-negative PTB patients was 48.7%, thus permitting these patients to start early and more adequate anti-TB treatment.

We further explored the factors associated with a positive Xpert MTB/RIF result in PTB children. These evaluated factors were (i) age; (ii) reported history of TB contact; and (iii) BCG vaccination. Firstly, interestingly, we found that age <3 years was independently associated with a positive result of Xpert MTB/RIF assay in PTB children. Traditionally, younger children are inclined to develop the paucibacillary primary disease, and indeed, and in contrast to our results, the age >5 years was previously shown to be associated with a positive Xpert MTB/RIF assay [26]. This difference may be explained, at least partly, by different severity of the enrolled PTB children or suggested that Xpert MTB/RIF assay can better detect TB patients in younger children less than 3 years old in our setting. Secondly, reported history of TB contact was also independently associated with a positive Xpert MTB/RIF assay in this study, which is in line with the previous report [26]. A reported history of TB contact increases the likelihood of suffering pulmonary TB in children. Thirdly, our results showed that the positive rate of Xpert MTB/RIF assay in unvaccinated children (without BCG scar) was significantly higher than in children with BCG scar. Long time ago, Rodrigues et al. confirmed that BCG vaccination can protect children from severe TB like miliary TB [27] and such patients may have high MTB load. This might explain why lack of BCG vaccination is associated with a positive Xpert MTB/RIF assay result in children.

In this study, Xpert MTB/RIF assay successfully identified one MDR-TB case. Our results, to some extent, verified the theory that Xpert MTB/RIF assay can be used to detect MDR-TB [8]. Traditional DST is based on culture, and it needs long time to diagnose MDR-TB, which will delay the treatment of patients with MDR-TB. In contrast, Xpert MTB/RIF assay providing RIF-resistance results within 2 hours is crucial to help clinicians to diagnose MDR-TB in children earlier and faster.

Also, this study has some limitations. Firstly, only relatively complicated patients with a chest radiograph who were suggested to do routine fiberoptic bronchoscopy were enrolled. And thus it may bring some bias. Secondly, limited by the volume of samples, we did one-sample test and thus may underestimate the diagnostic value of all methods. Thirdly, the finding of this study may not be widely applicable given that very few settings would do fiberoptic bronchoscopy.

## 5. Conclusion

Xpert MTB/RIF assay using BALF demonstrated an additional diagnostic value compared with AFB microscopy and MTB culture. It can assist in fast diagnosing childhood PTB when fiberoptic bronchoscopy is necessary according to the chest radiograph.

## Figures and Tables

**Table 1:** Characteristics of enrolled children with suspected PTB.

Characteristic	Frequency (*n*)	Percentage (%)
Gender		
Female	114	44.7
Male	141	55.3
Age (years old)		
~3	59	23.1
>3	196	76.9
BCG scar		
Absent	43	16.9
Present	212	83.1
Reported history of TB contact		
Yes	27	10.6
No	228	89.4

**Table 2:** Accuracy of Xpert MTB/RIF assay, culture, and smear on childhood PTB compared with CCRS.

Tests	Sensitivity *n*/*n*, %	Specificity *n*/*n*, %	PPV %	NPV %
Xpert MTB/RIF assay	44/83, 53.0 (41.8–63.9)	168/168, 100 (97.2–100)	100 (90.0–100)	81.2 (75.0–86.1)
MTB culture	24/83, 28.9 (19.7–40.1)	168/168, 100 (97.2–100)	100 (82.8–100)	74.0 (67.7–79.5)
AFB microscopy	7/83, 8.4 (3.7–17.1)	168/168, 100 (97.2–100)	100 (56.1–100)	68.8 (62.6–74.5)

**Table 3:** Accuracy of Xpert MTB/RIF assay on different kinds of childhood PTB.

Xpert MTB/RIF assay	Confirmed PTB *n* (%)	Probable PTB *n* (%)	Smear-positive PTB *n* (%)	Smear-negative PTB *n* (%)
Positive	24 (100)	20 (33.9)	7 (100)	37 (48.7)
Negative	0	39 (66.1)	0	39 (51.3)

**Table 4:** Factors associated with positive rate of Xpert MTB/RIF assay in children.

Factors	Xpert MTB/RIF assay		OR (95% CI)	*P* value
	Positive *n* (%)	Negative *n* (%)		
Gender				
Female	19 (16.8)	94 (83.2)	0.877 (0.402–1.911)	0.741
Male	25 (18.1)	113 (81.9)		
Age (years)				
~3	27 (48.2)	29 (51.8)	6.835 (3.099–15.074)	**<0.0001**
>3	17 (8.7)	178 (91.3)		
BCG scar				
Present	27 (12.8)	184 (87.2)	0.226 (0.096–0.531)	**0.001**
Absent	17 (42.5)	23 (57.5)		
Reported history of TB contact				
Yes	14 (53.8)	12 (46.2)	3.701 (1.364–10.04)	**0.010**
No	30 (13.3)	195 (86.7)		
